# The Relation Between Empathy and Aggression: The Role of Attachment Style

**DOI:** 10.5964/ejop.4509

**Published:** 2022-08-31

**Authors:** Maria Grazia Lo Cricchio, Pasquale Musso, Alida Lo Coco, Rosalinda Cassibba, Francesca Liga

**Affiliations:** 1 Department of Humanities, University of Studies of Basilicata, Potenza, Italy; 2 Department of Educational Sciences, Psychology, Communication, University of Studies of Bari “Aldo Moro”, Bari, Italy; 3 Department of Psychology, Educational Science and Human Movement, University of Studies of Palermo, Palermo, Italy; 4 Department of Clinical and Experimental Medicine, University of Studies of Messina, Messina, Italy; Edinburgh Napier University, Edinburgh, United Kingdom

**Keywords:** empathy, aggression, attachment style, profile, moderation

## Abstract

This study aimed to examine the explaining and moderating role of attachment style profiles on the association between empathy and aggression. Participants were 548 Italian adults (M = 47.62 years, SD = 6.14) who completed a survey measuring attachment, empathy, and aggression. Using cluster analytic methods, initial results indicated two attachment style profiles to be considered (secure vs. insecure). However, we also extracted a more theoretically guided four-cluster solution including preoccupied, secure, fearful, and dismissing profiles. Moreover, structural equation modelling showed that higher levels of empathy linked to lower levels of aggression. Nonetheless, when introducing in the model the dichotomous or the multi-categorical attachment style profile variable as predictive of both empathy and aggression, their association became not significant, while secure attachment profile significantly presented higher levels of empathy and lower levels of aggression compared to the other profiles. Furthermore, attachment style profile moderated the link between empathy and aggression. Specifically, in the secure group empathy and aggression were negatively related, but no significant association was evidenced in the other groups. Findings are discussed in the light of the literature.

Aggression among individuals refers to the intention to harm another person, while aggressive behaviour is the conduct that follows it (Anderson & Bushman, 2002). It is a central public health and societal problem that may produce direct physical damage and psychological and behavioural difficulties. Aggression may be expressed in many life aspects and contexts, and it is not only a problem for victims, but also for the aggressors, especially in adulthood: aggressive adults often show psychological problems, illegal behaviour, poor marital relationships, and unemployment (Coccaro, Noblett, & McCloskey, 2009). The strong effect of aggression on psychosocial adjustment underlines the need to recognize variables that can predict aggressive behaviour and that make some populations less or more susceptible to its perpetration. Awareness of such variables is fundamental for designing effective programmes for prevention, so a growing body of research has pursued the aim of understanding the processes and the personal characteristics that can reduce aggression, suggesting the potential role of empathy (e.g., Vachon, Lynam, & Johnson, 2014).

It is widely assumed that there is a connection between aggression and low empathy and that a lack of empathic responsiveness may be one of the main causes of aggression (van Hazebroek, Olthof, & Goossens, 2017). However, despite the general agreement on the association between these two constructs, several systematic reviews, and meta-analyses during the last years have found no or weak correlation between them (Bush, Mullis, & Mullis, 2000; Jolliffe & Farrington, 2004). These puzzling results call for new theoretical justifications, such as considering the explaining and moderating role of variables, such as attachment style, which has historically been considered as one of the stronger predictors of both empathy and aggression (Mikulincer & Shaver, 2001).

## The Relation Between Empathy and Aggression

Empathy refers to the ability to take the psychological point of view of another person, to understand and vicariously experience his/her thoughts and feelings (Davis, 2018). There is a consensus among scholars about its key role in the reduction of aggressive behaviour (Jolliffe & Farrington, 2004). Individuals that adopt the perspective of others and feel sympathy and compassion for them are more likely to refrain from behaving in ways that may cause harm to other people (Eisenberg, 2000). Bush and colleagues (2000) found that individuals who show, even occasionally, a higher level of aggressive behaviour, express lower empathic concern.

According to Gini, Albiero, and Benelli (2005), empathy can reduce aggressive manifestations by two main mechanisms: role-taking, which in ambiguous situations allows individuals to understand each other and to comprehend their reciprocal intentions and motivations; and perspective-taking, in which the vicarious sharing of the same emotions discourages mutual aggression. Farrington (1998) suggested that empathic responsiveness reduces aggressive behaviour because, especially in situations of conflict, it fosters the ability to understand the real motivations of others’ behaviours, reducing the risk of identifying as aggressive those conducts that do not have this nature or, where they have this connotation, enhancing the ability to tolerate them.

Despite these considerations, studies have not always confirmed these results. For example, Lindsey, Carlozzi, and Eells (2001) found no differences in empathy levels between violent and non-violent youths. Similar results were obtained by Goldstein and Higgins-D’Alessandro (2001) on groups of adults. Moreover, as previously mentioned, the same weak association between empathy and aggression has been well documented in recent meta-analyses and systematic reviews (Vachon, Lynam, & Johnson, 2014). Vachon et al. (2014) suggested that their relationship could vary depending on the type of moderating variables considered, such as age, gender, ethnicity, or level of education of the participants. However, they did not mention the need to consider attachment style, even though it has been shown to have a meaningful role in both empathy and the ability to manage aggressive behaviours.

## The Role of Attachment Style

Attachment theory (Bowlby, 1969) is one of the most important frameworks considered by researchers to investigate and explain the differences in individuals’ behaviours. According to Bowlby’s theory (1969), children’s relationships with their caregivers play an important role in the individuals’ social and emotional development. The ability to relate to others appropriately, sharing emotions with them and understanding their points of view, is the consequence of childhood relationships with one or more caregivers defined as “sensitive” (Ainsworth, Blehar, Waters, & Wall, 1978), which enable the development of a secure attachment bond. This type of relationship is established as an expression of a deep and warm emotional relationship with the caregiver: from repeated experiences with sensitive caregivers, children develop confidence in their availability as secure bases, which allow them to explore the environment counting on their help and protection, in case of danger (Srivastava & Beer, 2005). These feelings of trust and support, however, are missing in some individuals, who, in contrast, develop an insecure attachment style, based on a relationship in which affective needs were not satisfied (Saltaris, 2002).

The first relational experiences are the basis for the development of Internal Working Models (IWM), representations of themselves, and others, which generally guide individuals’ behaviours during development (Pietromonaco & Barrett, 2000). In the case of secure attachment, IWM involve a positive representation of self and others: adults with a secure attachment bond are more inclined to trust others and expect positive intentions from them (Dodge, 2006). These positive IWM can explain why adult attachment security has been frequently related with higher levels of empathy and lower personal distress (Mikulincer, Shaver, Gillath, & Nitzberg, 2005). Concerning insecure attachment, Bartholomew and Horowitz (1991) distinguished three prototypically different styles depending on their IWM of self and others: preoccupied, fearful, and dismissing. Preoccupied people have a positive view of others, but a negative view of self and they are often searching for others’ approval. Fearful people have negative views of themselves and others, and feel unloved, expecting to be rejected by others. Dismissing people have a positive view of self and a negative view of others, and they are rejecting other people to maintain their high sense of self (Bartholomew & Horowitz, 1991; Feeney & Noller, 1990). Literature has broadly underlined that insecurely attached people are susceptible to experiencing negative emotions due to the rejecting or inconstant responses offered by their caregivers (Grattagliano et al., 2015; Troyer & Greitemeyer, 2018). Moreover, they might show more self-centred emotional reactions and difficulties in considering the real emotions and needs of others (van der Mark, van Ijzendoorn, & Bakermans-Kranenburg, 2002). As such, attachment insecurity can distort or inhibit empathic responsiveness (Feeney & Collins, 2001). For example, the extreme worries of rejection that characterize highly preoccupied individuals might leave them with little cognitive resources to emotionally share others’ distress (Mikulincer & Shaver, 2007). Additionally, highly fearful and dismissing people may be less likely to express empathy as a consequence of their mistrust in others’ support and help in case of need: a lack of empathy may be a way to maintain others at distance, preventing them from becoming too close, and precluding potential reliance and dependence (Mikulincer & Shaver, 2007).

Even the ability of self-regulation of aggressive behaviour is closely related to the type of attachment bond. In this sense, an individual experiencing security and trust in relationships with others would more probably be a person not inclined to aggression, because he/she does not see dangers and threats in the people he/she meets (Mikulincer & Shaver, 2001). On the other hand, fearful and dismissing individuals may become aggressive to keep others at distance and avoid intimacy because they have learned that closeness means being upset and rejected (negative model of others) (Péloquin, Lafontaine, & Brassard, 2011). Moreover, they might develop a mental model that enhances the likelihood of thinking that others may reject or damage them, diminishing their ability to evaluate situations in an objective manner (Thompson, 2008). Contrariwise, preoccupied individuals wish complete intimacy with others, but, since they are extremely concerned with their lovability (negative model of self), they might have fears of remaining alone (Péloquin et al., 2011). Consequently, they would use more likely aggression toward others to express their irritated and frustrated needs of closeness in the relationship.

Despite this evidence, to our knowledge, no studies seem to have explored whether and how attachment style affects the association between empathy and aggression. However, as mentioned, it is theoretically sustainable that this relationship can vary according to the type of attachment bond. Specifically, individuals with a secure attachment are more likely to understand and share others’ emotional states and, therefore, are more likely to have psychological resources and instruments that can be useful to manage and to inhibit their aggressive behaviours generating concern for the well-being of others. As a result, a secure attachment style may strengthen the negative association between empathy and aggression. In contrast, insecurely attached individuals seem to live their life with a lower capacity to share desires, needs, and emotions with others. On the one hand, it is more likely that other people’s negative emotions can cause high emotional distress to preoccupied people (Davis, 2018) and potential aggressive motivations/behaviours to reduce this perceived discomfort. On the other hand, insecure fearful or dismissed individuals might be less interested in other emotions, showing low or even no willingness to keep in contact with them, which can enhance their aggressive behaviour to maintain others at distance (Eisenberg, Shea, Carlo, & Knight, 1991). In this sense, for insecurely attached people, the negative association between empathy and aggressive behaviour could be lower. Accordingly, our study had the main and original goal to investigate how attachment style can influence the relations between empathy and aggression.

Moreover, the methodology adopted may limit the existing knowledge on the relation between these constructs in that most of the studies have exclusively used a variable-centred approach in their examinations that may not be optimal for capturing their multi-composite nature. In fact, variable-centred methods are aimed at describing differences in the mean-level and/or the linear associations of unique dimensions, whereas the person-centred approach emphasizes the need to understand the individual as a whole, rather than on his/her single characteristics (Bergman, 2001). Within the broad debate about the best approach to measure and conceptualize attachment (e.g., Fraley, Hudson, Heffernan, & Segal, 2015), our perspective is that the person-centred approach best reflects the goal of grasping attachment styles by assigning individuals sharing similar patterns of attachment-related variables to subgroups within a given population: this holistic and dynamic view emphasizes the potential uniqueness of individuals and allows to obtain profiles which, then, can be compared on several correlates to detect interindividual differences. Therefore, our second aim was to investigate the specific differences in the relations between empathy and aggression arising from the diverse configurations of attachment style, by using a mixed person- and variable-oriented approach.

## Method

The current study aimed at investigating the explaining and moderating role of attachment style on the associations between empathy and aggression. First, we studied whether the attachment style can explain the expected negative association between empathy and aggression within a variable-centred approach. Second, we examined how different attachment style profiles may influence the link between empathy and aggression within a mixed person- and variable-centred approach. Specifically, we hypothesized that: (a) empathy and aggression are negatively related; (b) when introducing the attachment style dimension as an explaining variable of both empathy and aggression, their association will be lower due to the important predictive role of this dimension; and (c) a more securely attached profile group will show a stronger negative association between empathy and aggression than insecurely attached profile groups, due to higher level of psychological resources of secure individuals that can inhibit one's aggressive behaviours by having concern and interest for the others' welfare.

### Participants

Five hundred and forty-eight adults (64% females and 36% males) aged 36 to 63 years (*M* = 47.62, *SD* = 6.14), living in the urban area of the city of Palermo (Italy), participated in the study. The sample size was derived based on a theoretical correlation coefficient of *r* = -.113 between empathy and aggression, as suggested by the recent meta-analyses of Vachon et al. (2014). By establishing the threshold probability for rejecting the null hypothesis (σ two-tailed, Type I error rate) at .05 and the probability of failing to reject the null hypothesis under the alternative hypothesis (Type II error rate) at .20, the exact sample size was computed to be 612. However, we were able to collect a sample that was only 90% of this expected number but still acceptable (corresponding to a theoretical correlation coefficient of *r* = -.119). Participants were almost all Caucasian Italians (91%). Most of them (86%) were married, 10% were never married, 3% were separated/divorced, and 1% were widowed. In terms of educational level, 21% of the entire group had completed primary school level, 42% had finished middle school, 27% had obtained a high school diploma, and 10% had graduated. Additionally, 54% of the participants declared themselves as employed and the remaining 46% as unemployed (but 86% of this group presented themselves as homemakers). These demographic data were satisfactorily consistent with the general trends for middle-aged adults in Southern Italy, as reported by the Italian National Statistics Institute (ISTAT, 2018).

### Procedure

The ethical code of the Italian Association of Psychology (2015) was followed throughout the research process. Participants were equally recruited from each of the eight districts of the city of Palermo during public events open to a wide range of people, such as music and folkloristic festivals or exhibitions. On these occasions, the members of the research group distributed leaflets informing the public about the purpose of the study and asking for voluntary anonymous participation. Upon initial acceptance, interested persons were further made aware of the study and its implications by a written informed consent sheet, to be signed to give consent or not signed to indicate lack of consent. All the individuals who were initially available to contribute to the study agreed to the signed informed consent and, therefore, they were invited to fill in a self-administered questionnaire. They could withdraw at any time, but there were no withdrawals. On average, participants completed the questionnaire in 30 minutes.

### Measures

#### Socio-Demographics

Participants were asked to report their gender, age, ethnicity, marital status, level of school completed, and occupation.

#### Attachment

Different attachment dimensions were assessed by the Italian version of the Attachment Style Questionnaire (ASQ; Fossati et al., 2007). This self-report questionnaire asks participants to rate on a 6-point scale the degree to which they agree with 40 statements concerning their perceptions of themselves and their relationships (1 = *totally disagree* to 6 = *totally agree*). Specifically, ASQ measures: confidence (eight items; “I feel confident that other people will be there for me when I need them”), discomfort with closeness (10 items; “I find it difficult to depend on others”), relationships as secondary (eight items; “Achieving things is more important than building relationships”), need for approval (seven items; “It’s important to me that others like me”), and preoccupation with relationships (seven items; “I worry a lot about relationships”). The confidence scale, representing secure attachment, refers to the extent to which individuals are confident about themselves and their relationships with others. Each of the other four scales represents aspects of insecure attachment. Discomfort with closeness refers to the difficulty in trusting others, depending on other people, or having other people depend on them. Relationships as secondary refers to the belief that achievement is more important than relationships. The need for approval refers to the view that it is important to measure up to others’ standards and to be positively identified by other people. Finally, the preoccupation with relationships dimension refers to the anxiety of being abandoned because of the belief of being unable to cope alone. In the current study, Cronbach’s alpha (α) values were .78, .81, .70, .75, and .79 for confidence, discomfort with closeness, relationships as secondary, need for approval, and preoccupation with relationships, respectively.

#### Empathy

Empathy was assessed by the Italian version of the Interpersonal Reactivity Index (IRI; Albiero, Ingoglia, & Lo Coco, 2006). This self-report questionnaire asks participants to rate on a 5-point scale the degree to which they agree with 28 statements concerning the responsivity to others (1 = *never true* to 5 = *always true*). Specifically, IRI consists of four 7-item subscales: personal distress ("Being in a tense emotional situation scares me") and empathic concern ("I often have tender, concerned feelings for people less fortunate than me") being emotional aspects, and perspective-taking ("I sometimes try to understand my friends better by imagining how things look from their perspective") and fantasy ("I really get involved with the feelings of the characters in a novel") being cognitive aspects. The personal distress scale assesses the personal experience of feeling anxious and awkward in stressful interpersonal situations, and the empathic concern scale assesses feelings of understanding, consideration, and kindness for others experiencing negative events. The perspective-taking scale assesses the ability to assume the viewpoint of other people in everyday life, and the fantasy scale assesses the tendencies to be imaginatively moved by feelings and actions of invented characters in books, movies, and plays. Cronbach’s α values for the present study were .72 for personal distress, .70 for empathic concern, .76 for perspective-taking, and .73 for fantasy. However, we excluded fantasy from the analyses because this construct is less consistent with the definition of empathy and, for this reason, it was often overlooked in studies (e.g., Davis, Hall, & Meyer, 2003).

#### Aggression

Aggression was assessed using the Irritability and Rumination/Dissipation Questionnaire (I-RQ; Caprara, Barbaranelli, Pastorelli, & Perugini, 1991). This self-report questionnaire asks participants to rate on a 7-point scale their reaction to 20 statements concerning emotive-impulsive and cognitive dimensions of aggression behaviour (1 = *completely false for me* to 7 = *completely true for me*). This questionnaire consists of two 15-item scales, each composed of 10 items supplemented by 5 additional items used to check compliance: irritability (“When someone raises his voice, I raise mine higher”) and rumination/dissipation (“When I am outraged, the more I think about it, the angrier I feel”). The irritability scale measures the emotive processes affecting aggressive behaviour, which is the tendency to react impulsively, argumentatively, or rudely at the slightest provocation or disagreement. The rumination/dissipation scale assesses the cognitive processes affecting aggressive behaviour, that is respondents’ tendencies to hold grudges and to hold thoughts of retaliation, in addition to difficulty in forgetting injustices that have been committed towards them. Cronbach’s α values for the present study were .75 for irritability and .85 for rumination/dissipation.

#### Plan of Analysis

Data analysis followed three main steps. First, we computed descriptive statistics for the key study variables and verified for univariate and multivariate normality.

Second, we utilized a cluster analytic approach to identify attachment style patterns based on the standardized scores of the ASQ subscales. Initially, agglomerative hierarchical cluster analyses were carried out to determine the most appropriate number of clusters. Using Ward’s method based on the squared Euclidean distance (Aldenderfer & Blashfield, 1984), solutions from two to five clusters were assessed. The best number of clusters was chosen as a balance among theoretical meaningfulness, parsimony, and explanatory power (i.e., the proportion of the variance explained in each ASQ dimension). After that, study participants were grouped by *K*-means cluster analysis procedures. To check the validity of the solution, a multivariate analysis of variance (MANOVA) on the five ASQ dimensions by cluster was performed. We also tested the replicability of the solution by randomly dividing the data into two samples (A and B) and performing a full cluster analysis on each sample. Sample B was then classified into clusters according to the cluster centres derived from Sample A, and the agreement between the two Sample B solutions was computed using Cohen’s kappa, with higher agreement indicative of a more stable cluster solution.

Third, once participants were classified, we explored the explaining and moderating role of attachment style on the association between empathy and aggression using a structural equation modelling (SEM) approach. We initially tested the relationship between the latent variable with three indicators of empathy (personal distress, empathic concern, and perspective-taking) and the latent variable with two indicators of aggression (irritability and rumination/dissipation). This relationship was modelled in terms of a direct pathway from empathy to aggression. Gender (0 = male; 1 = female), age, marital status (dummy coded: 0 = not cohabiting with a partner; 1 = cohabiting with a partner), and school level (dummy coded: 0 = up to middle school; 1 = high school and beyond) were controlled for. Then, we tested the explaining and moderating models. The explaining model included the attachment style profile variable in the initial model to analyse its influence on the previous relationship between empathy and aggression. In this case, we modelled the new associations adding direct pathways from attachment style profile to both empathy and aggression. The moderating model corresponded to a multiple-group SEM with attachment style profile as the grouping variable. Following Kline (2015), multiple indices and their associated cut-offs were used to evaluate model fit: the chi-square (χ^2^) test value with *p* > .05 or close to .05, CFI ≥ .90 for acceptable and ≥ 0.95 for good fit, RMSEA ≤ .08 for acceptable and ≤ .05 for good fit, and SRMR ≤ .10 for acceptable and ≤ .05 for good fit. Given the different number of variables, fit comparison between the initial and explaining (non-nested) models was performed using the Akaike Information Criterion (AIC; Burnham & Anderson, 2004). A better fitting model would significantly have had a lower AIC value when the following criterion was ascertained: ΔAIC ≥ 15.00 as an absolute value (Viola, Musso, Ingoglia, Lo Coco, & Inguglia, 2017). Nested model comparisons for the more restrictive vs. the less restrictive multi-group models were used to examine whether the path between empathy and aggression differed by attachment style profile. To ascertain significant differences at least two out of these three criteria had to be satisfied: Δχ^2^ significant at *p* < .05, ΔCFI ≤ –.010, and ΔRMSEA ≥ .015 (Kline, 2015).

## Results

### Descriptive Statistics

Table 1 reports the descriptive statistics. All the variables were normally distributed with skewness and/or kurtosis values < |1.00| or close to this value as well as values of standardized scores < |3.29| (Tabachnick & Fidell, 2013). Multivariate inspection of the data, using Mahalanobis distance with *p* < .001 and Mardia’s multivariate kurtosis coefficient, evidenced four slight outliers. After replicating the analyses with or without these cases, no significant effects on the results were found. Thus, the initial sample was fully maintained.

**Table 1:** Means, Standard Deviations, Skewness, and Kurtosis for the Key Study Variables

Variable	*M*	*SD*	Skewness	Kurtosis
1. Confidence	4.28	.58	-.44	.11
2. Discomfort with closeness	3.81	.65	-.29	.25
3. Relationships as secondary	2.61	.88	.44	.23
4. Need for approval	2.89	.90	.11	-.37
5. Preoccupation with relationships	3.49	.74	-.41	.76
6. Personal distress	2.14	.52	.32	.41
7. Empathic concern	2.98	.34	-.32	-.62
8. Perspective taking	2.69	.43	.40	.34
9. Irritability	3.62	1.83	.34	-1.08
10. Rumination/Dissipation	4.05	1.85	-.09	-1.13

### Attachment Style Profiles

**Figure 1:**
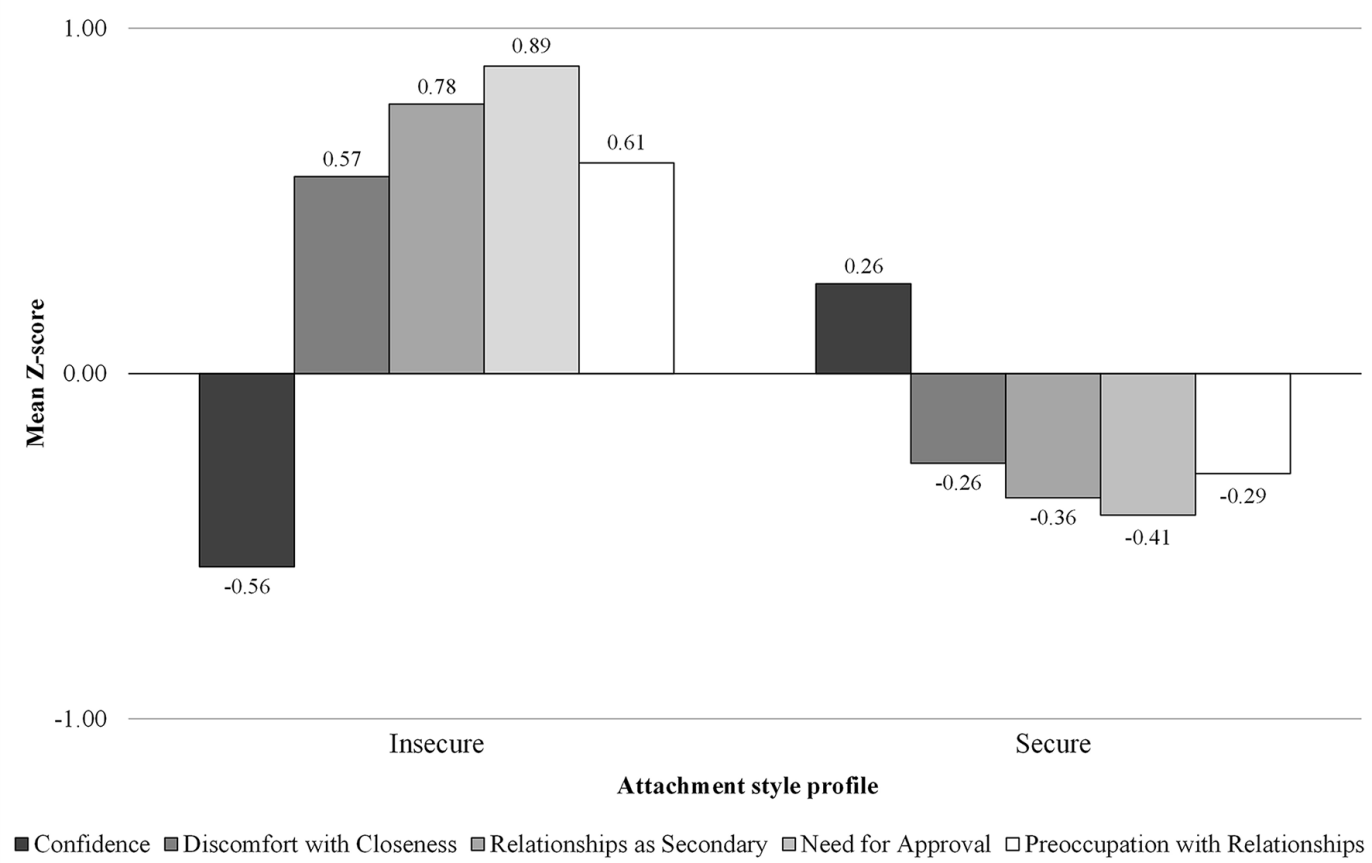
Mean Z-Scores for the Attachment Style Questionnaire Variables by the Two Attachment Style Profiles

Based on the initial agglomerative hierarchical cluster analyses, a two-cluster solution emerged as the most adequate. Generally, solutions with a greater number of clusters violated the principles of parsimony, explanatory power, and/or theoretical meaningfulness, including clusters that presented slight differences compared to the two clusters and were scarcely interpretable. Thus, participants were clustered into two groups by *K*-means cluster analysis. Figure 1 displays the emerged attachment style profiles. The first cluster (*n* = 174; 31.8%) was composed of adults who scored higher on discomfort with closeness, relationships as secondary, need for approval, and preoccupation with relationships, but lower on confidence. The second cluster (*n* = 374; 68.2%) consisted of adults scoring higher on confidence but lower on all the other variables. We respectively named these clusters as insecure and secure attachment style profiles.

The MANOVA performed on the ASQ variables by profile revealed a significant multivariate effect, Wilks’ Lambda = .40, *F*(5, 542) = 159.47, *p* < .001, partial η^2^ = .60, showing that 60% of the variability was explained by differences among the two clusters. Post-hoc tests of between-subjects’ effects revealed that the two-cluster solution explained 15% of variability for confidence, 15% for discomfort with closeness, 28% for relationships as secondary, 37% for need for approval, and 18% for preoccupation with relationships, as reported by partial eta square (η^2^). According to Richardson (2011), partial η^2^ effect size of greater than or equal to .1379 (that is, 13.79% of variability explained) is considered large; accordingly, the two-cluster solution explained large proportions of variance for each variable. Moreover, the replicability procedure confirmed that the two-cluster solution was the best also for both the two random subsamples and that the agreement value was substantial (*k* = .69).

**Figure 2:**
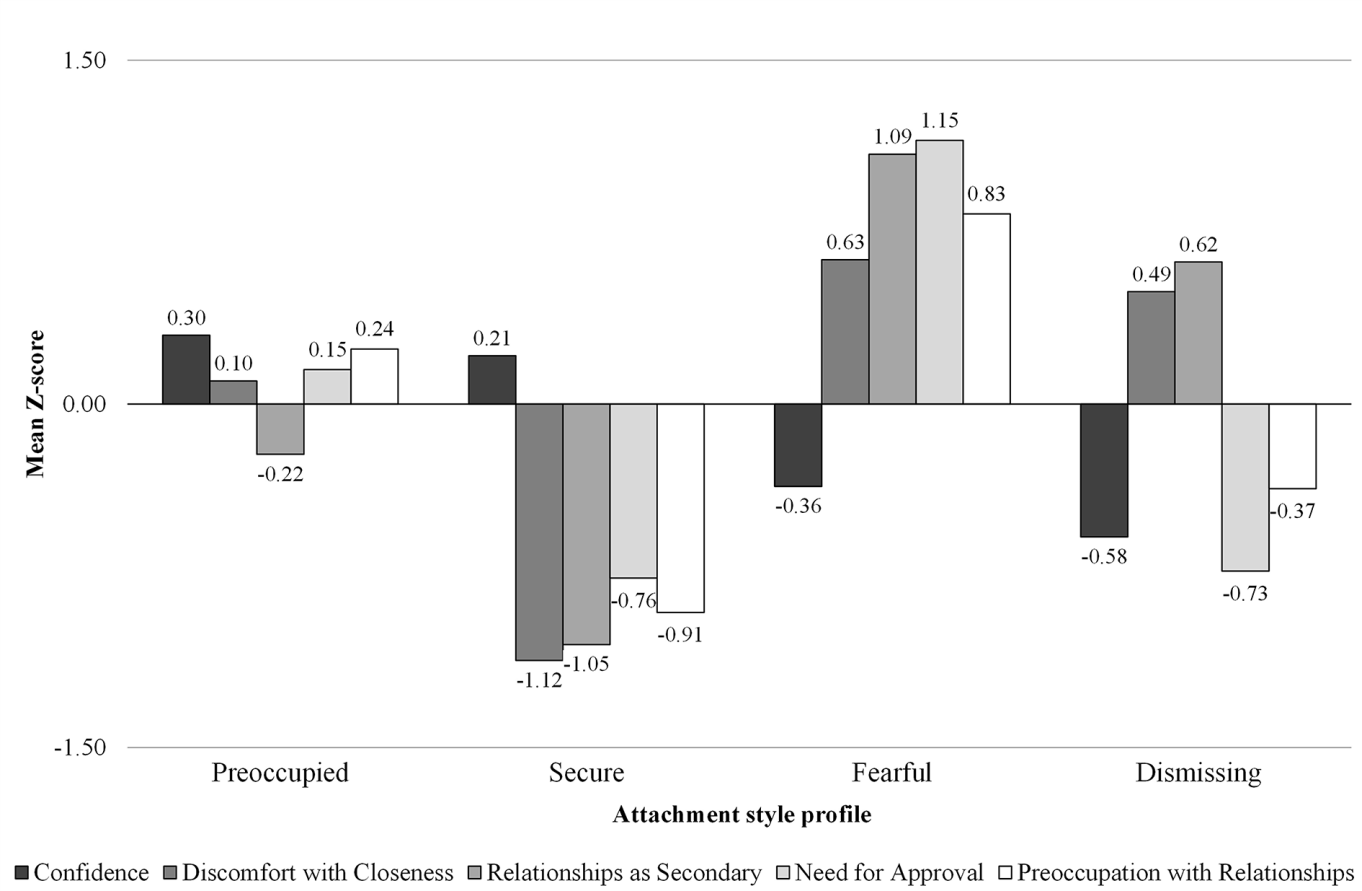
Mean Z-Scores for the Attachment Style Questionnaire Variables by the “Forced” Four-Cluster Solution of Attachment Style

In addition to this first set of analyses, we also conducted a new *K*-means cluster analysis of ASQ scores, in which we “forced” a four-cluster solution based on the results of prior studies indicating three (quite different) prototypically insecure attachment styles (see, for example, Bartholomew & Horowitz, 1991). Figure 2 shows the attachment style profiles obtained. The first cluster (*n* = 224; 40.9%) included participants who scored higher on confidence and preoccupation with relationships, moderately higher on need for approval and discomfort with closeness, but lower on relationships as secondary. The second cluster (*n* = 122; 22.2%) comprised adults scoring higher on confidence but lower on all the other variables. The third cluster (*n* = 110; 20.01%) consisted of adults scoring lower on confidence but higher on all the other variables. The fourth cluster (*n* = 92; 16.8%) was composed of adults who scored higher on relationships as secondary and discomfort with closeness, but lower on confidence, need for approval, and preoccupation with relationships. We respectively named these clusters as preoccupied, secure, fearful, and dismissing attachment style profiles, because they approximately resembled the attachment styles suggested by Bartholomew and Horowitz (1991).

The MANOVA performed on the ASQ variables by this four-profile solution revealed a significant multivariate effect, Wilks’ Lambda = .12, *F*(15, 1491) = 116.55, *p* < .001, partial η^2^ = .51, showing that 51% of the variability was explained by differences among the four clusters (less than 60% of the two-cluster solution). Post-hoc tests of between-subjects’ effects revealed that the four-cluster solution explained 13% of variability for confidence (below the cut-off for large effects suggested by Richardson, 2011), 40% for discomfort with closeness, 57% for relationships as secondary, 49% for need for approval, and 37% for preoccupation with relationships. Moreover, the replicability procedure revealed a Cohen’s *k* equal to .63, indicative of a just sufficiently stable solution. These results revealed that this theoretically guided four-cluster solution was not perfectly adequate, but still acceptable. Therefore, we used it as a comparison to the more optimal two-cluster solution in subsequent analyses.

### Association Between Empathy and Aggression

Table 2 shows bivariate correlations among the key and control study variables. The attachment style profile is presented both as a dichotomous (0 = insecure; 1 = secure) and a multi-categorical (preoccupied, secure, fearful, and dismissing) variable. In this latter case, we used a dummy coding strategy to represent the groups in the correlation matrix and the subsequent models. The secure attachment style profile was not explicitly coded; that is, all the *k*-1 dummy variables (*k* denotes the total number of categories) were set to 0 for cases in that group. Thus, the secure attachment style profile functioned as the reference group in the analyses, and coefficients and parameters interpreted relative to this reference group.

**Table 2:** Correlations For Key and Control Study Variables

Variable	1	2	3	4	5	6	7	8	9	10	11	12	13
1. Attachment style profile (0 = insecure; 1 = secure)	—												
2. Preoccupied vs. secure attachment profile	.22***	—											
3. Fearful vs. secure attachment profile	-.73***	-.42***	—										
4. Dismissing vs. secure attachment profile	.10*	-.37***	-.23***	—									
5. Personal distress	-.08	.01	.12**	.00	—								
6. Empathic concern	.09*	-.05	.02	-.13**	-.20***	—							
7. Perspective taking	.10*	-.02	-.09*	-.06	-.26***	.32***	—						
8. Irritability	-.30***	.00	.23***	.07	.04	-.07	-.09*	—					
9. Rumination/Dissipation	-.08*	.02	.10*	.15***	.04	-.06	-.06	.40***	—				
10. Gender (0 = male; 1 = female)	.03	.08	-.04	-.17***	.04	.03	.03	-.29***	-.19***	—			
11. Age	.08	.02	-.09*	.02	-.05	.05	.09*	.08	.13**	-.24***	—		
12. Marital status (0 = not cohabiting; 1 = cohabiting with partner)	-.05	-.10*	.07	-.05	.05	.03	-.03	.06	.08	-.10*	-.06	—	
13. School level (0 = up to middle school; 1 = high school and beyond)	.07	-.09*	-.05	-.02	-.11**	.06	-.03	-.22***	-.12**	.07	.08	-.35***	—

*p < .05. **p < .01. ***p < .001.

**Figure 3:**
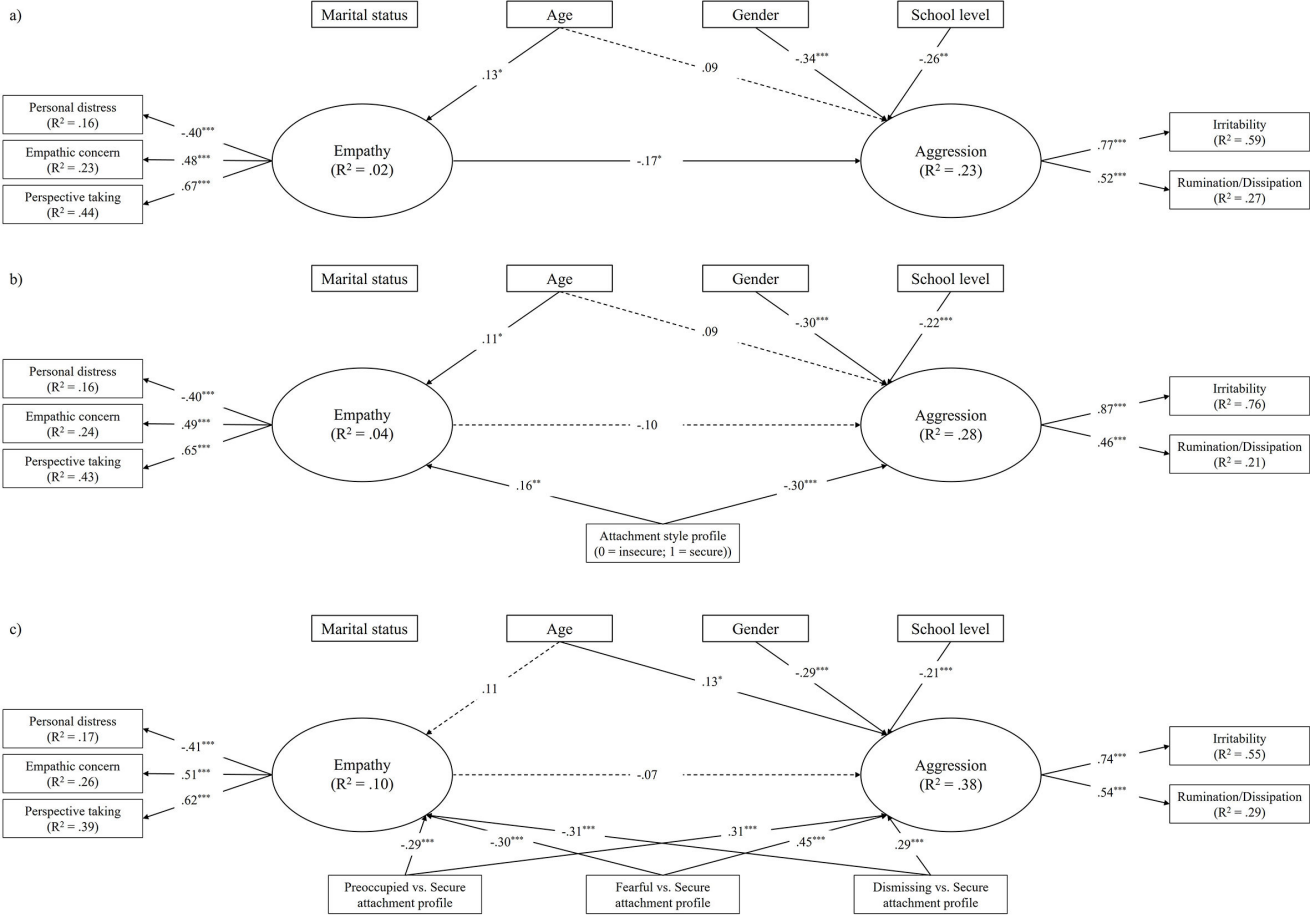
Estimated Structural Equation Models for the Empathy-Aggression Relation Before and After Including the Dichotomous and Multi-Categorical Attachment Style Profile Variable *Note.* Maximum likelihood standardized coefficients are shown. Correlations among control variables and residuals are not shown for brevity. Solid lines represent significant pathways, dashed lines are nonsignificant. Pathways not represented are fixed to zero. *p < .05. **p < .01. ***p < .001.

We estimated the initial model by testing the association of the latent variable of empathy with the latent variable of aggression. We controlled for gender, age, marital status, and school level by allowing them to predict both the latent variables; however, only the parameters with *p* ≤ .10 were finally estimated while the others were fixed to zero. This model fitted the data well, χ^2^(20) = 25.60, *p* = .18, CFI = .981, RMSEA = .023, 90% CI [.000, .046], SRMR = .025, AIC = 7,517.34. As shown in Figure 3a, empathy was significantly and negatively associated with aggression. Additionally, age was significantly and positively linked to empathy (the higher the age, the higher the empathy), while gender and school level were only significantly and negatively linked to aggression (females showed lower aggression as well as the higher the school level, the lower the aggression).

When including the dichotomous attachment style profile variable, the new model also had a good fit, χ^2^(23) = 31.06, *p* = .12, CFI = .976, RMSEA = .025, 90% CI [.000, .046], SRMR = .025, AIC = 7,468.59, indeed a better fit compared to the initial model, ΔAIC = 48.75 (see Figure 3b). Results showed that the secure attachment style profile was significantly and positively related to empathy, while it was significantly and negatively linked to aggression (the opposite for the insecure attachment style profile). Also, the association between empathy and aggression was not significant (β = -.10, *p* = .11), supporting the evidence that the initial significant association could be fully explained by their common relationship with the attachment style profile. Control variables showed the same pattern of associations with empathy and aggression as for the initial model.

Furthermore, we tested a model that included, instead of the dichotomous, the multi-categorical attachment style profile variable through the dummy variables of preoccupied vs. secure profile, fearful vs. secure profile, and dismissing vs. secure profile. The model fit was acceptable, χ^2^(29) = 46.04, *p* = .02, CFI = .955, RMSEA = .033, 90% CI [.012, .050], SRMR = .025, AIC = 7,446.01, and had a better fit compared to the initial model, ΔAIC = 71.33 (see Figure 3c). Results showed that the three dummy variables were significantly and negatively related to empathy as well as significantly and positively related to aggression. That is, participants in the preoccupied, fearful, and dismissing attachment profiles showed lower empathy and higher aggression compared with participants in the secure attachment profile. As in the previous model including the dichotomous attachment style profile variable, the association between empathy and aggression was not significant (β = -.07, *p* = .30) and control variables showed the same pattern of associations with empathy and aggression except for age which was significantly and positively linked to aggression and not to empathy. We also replicated the analysis using, in turn, the preoccupied, the fearful, and the dismissing attachment profiles as the reference group for the dummy variables. While no significant differences were found between participants in these three different profiles concerning empathy, participants in the fearful attachment profile showed higher aggression compared with those in the preoccupied and dismissing attachment profiles (respectively, β = .20, *p* < .001, and β = .26, *p* < .05, when the preoccupied and the dismissing attachment profiles were the reference group).

### The Moderating Model

**Figure 4:**
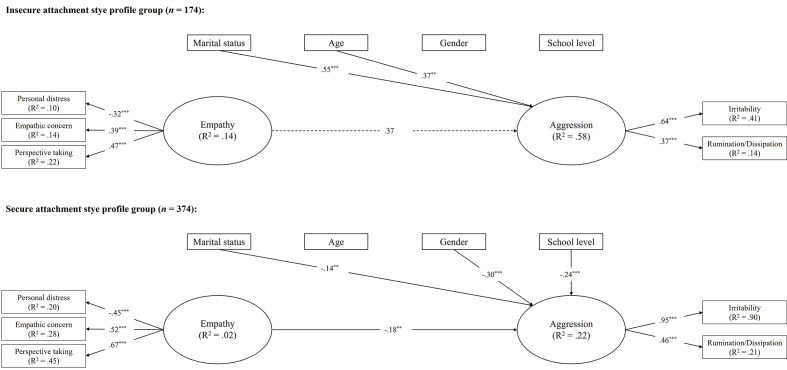
Estimated Multiple-Group Structural Equation with the Dichotomous Attachment Style Profile as the Grouping Variable *Note.* Maximum likelihood standardized coefficients are shown. Non-significant pathways for control variables, correlations among control variables, and residuals are not shown for brevity. Solid lines represent significant pathways, dashed lines are nonsignificant (*p* > .05). The estimate of the empathy-aggression pathway in the insecure attachment style profile group was nonsignificant due to the high standard deviation value (.194). **p < .01. ***p < .001.

First, we considered the multiple-group SEM with the dichotomous attachment style profile as the grouping variable. Table 3 reports the bivariate correlations. An initial model was run so that all the pathways, and especially that from empathy to aggression, were freely estimated, while all factor loadings were constrained to be equal across the two attachment style profile groups (secure vs. insecure). The model had adequate fit, χ^2^(38) = 53.52, *p* = .05, CFI = .952, RMSEA = .039, 90% CI [.003, .061], SRMR = .036. Next, we also constrained the pathway from empathy to aggression, obtaining a significantly worse fit, Δχ^2^(1) = 7.24, *p* = .007, ΔCFI = - .019, ΔRMSEA = .006. This suggested that the association between empathy and aggression was moderated by the attachment style profile as seen in Figure 4. Particularly, empathy was significantly and negatively related to aggression in the secure attachment style group, but not in the insecure one (β = .37, *p* = .06). Also, in the insecure attachment style group, higher age and cohabiting with a partner were significantly associated with higher aggression, while in the secure one being female and cohabiting with a partner as well as higher school level were linked to lower aggression.

**Table 3:** Correlations for Study Variables by Dichotomous Attachment Style Profile

Variable	1	2	3	4	5	6	7	8	9
1. Personal distress	—	-.27***	-.30***	.08	.05	.03	-.04	.08	-.15**
2. Empathic concern	.01	—	.33***	-.11*	-.11*	.08	-.03	.05	.05
3. Perspective taking	-.14	.26***	—	-.15**	-.11*	.10	.07	-.03	-.03
4. Irritability	-.12	.12	.13	—	.45***	-.33***	.04	-.06	-.24***
5. Rumination/Dissipation	-.01	.12	.09	.26***	—	-.19***	.12*	.03	-.15**
6. Gender	.08	-.08	-.14	-.23**	-.18*	—	-.18***	-.05	.13*
7. Age	-.07	.22**	.11	.25**	.19*	-.38***	—	.00	.06
8. Marital status	-.06	-.02	-.02	.29***	.18*	-.21**	-.20**	—	-.27***
9. School level	.02	.05	-.05	-.15*	-.03	-.07	.10	-.52***	—

*Note*. Lower diagonal: correlation matrix for insecure attachment style group. Upper diagonal: correlation matrix for secure attachment style group.

*p < .05. **p < .01. ***p < .001.

**Figure 5:**
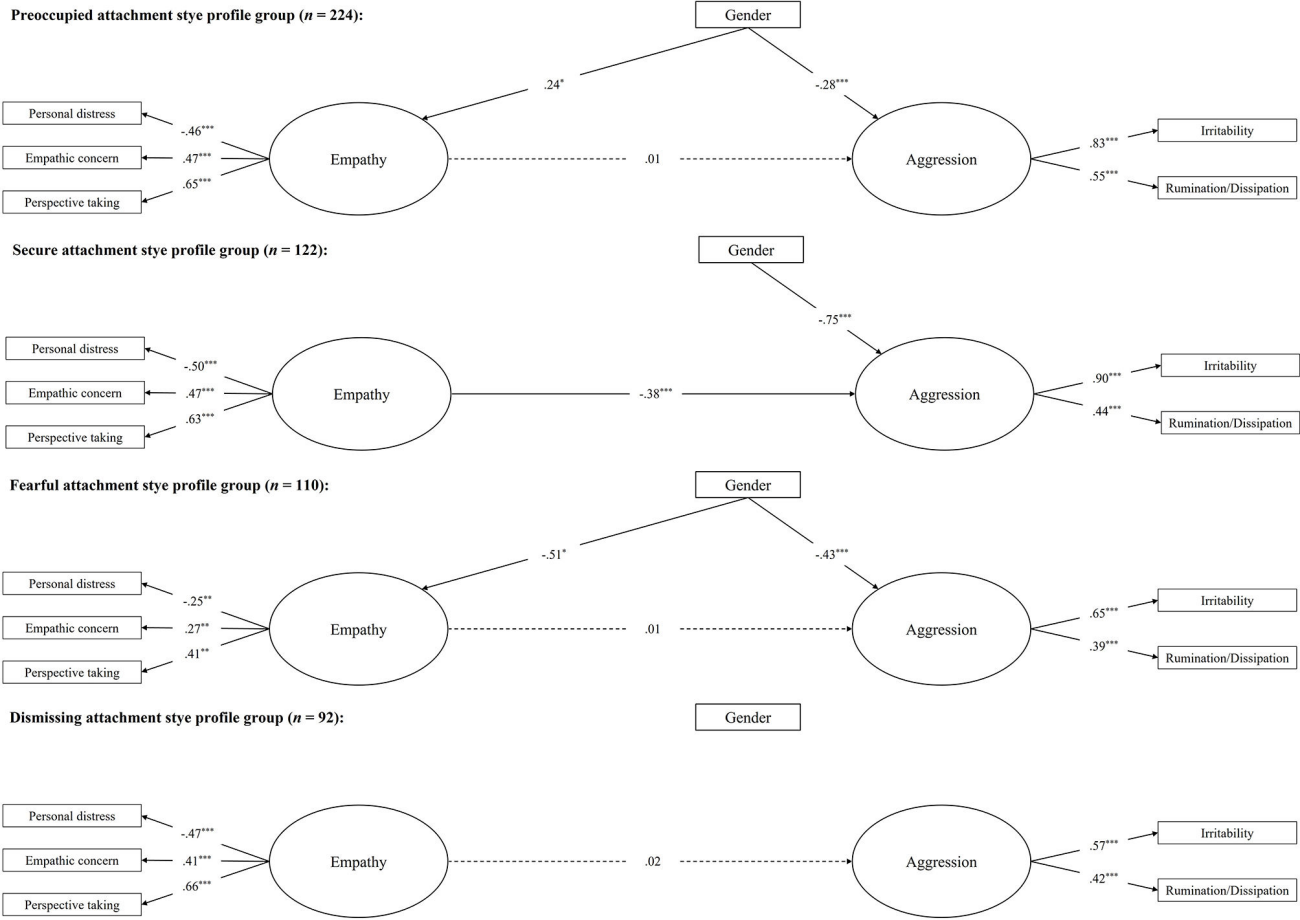
Estimated Multiple-Group Structural Equation With the Multi-Categorical Attachment Style Profile as the Grouping Variable *Note.* Maximum likelihood standardized coefficients are shown. Non-significant pathways for gender and residuals are not shown for brevity. Solid lines represent significant pathways, dashed lines are nonsignificant. *p < .05. **p < .01. ***p < .001.

Second, we repeated the analysis using the multi-categorical attachment style profile as the grouping variable. Table 4 reports the bivariate correlations. The initial model, with only the factor loadings constrained to be equal across the four attachment style profile groups, had a bad fit, χ^2^(82) = 182.70, *p* < .001, CFI = .775, RMSEA = .095, 90% CI [.076, .113], SRMR = .070. Additional analyses suggested excluding the control variables of age, marital status, and school level to ensure a more adequate fit. Thus, we re-ran the model by including only gender among the control variable. The new model showed an acceptable fit, χ^2^(46) = 72.81, *p* = .01, CFI = .904, RMSEA = .065, 90% CI [.034, .093], SRMR = .063. The nested model constraining the pathway from empathy to aggression across all groups resulted in a significantly worse fit, Δχ^2^(3) = 8.00, *p* = .046, ΔCFI = - .015, ΔRMSEA = .003. Modification indices suggested to freely estimate the empathy-aggression link for the secure attachment profile group. The resulting partial constrained model was sufficiently supported when compared to the initial model, Δχ^2^(2) = 0.43, *p* = .80, ΔCFI = .006, ΔRMSEA = -.003. Again, this suggested the moderating role of the attachment style profile in the relation between empathy and aggression, with empathy significantly and negatively related to aggression in the secure attachment style group, but with no significant relation to aggression in any of the other groups (see Figure 5). Also, being female was significantly linked to lower aggression in the preoccupied, secure, and fearful groups, and was significantly associated with higher empathy in the preoccupied group but with lower empathy in the fearful group.

**Table 4:** Correlations for Study Variables By Multi-Categorical Attachment Style Profile

Variable	1	2	3	4	5	6	7	8	9
Preoccupied profile									
1. Personal distress	—								
2. Empathic concern	-.22**	—							
3. Perspective taking	-.25***	.35***	—						
4. Irritability	-.09	-.08	-.06	—					
5. Rumination/Dissipation	-.06	-.09	-.10	.45***	—				
6. Gender	-.11	.08	.17**	-.23***	-.13	—			
7. Age	-.01	.03	.01	.13	.17*	-.10	—		
8. Marital status	.17*	.08	-.01	.06	-.11	-.21**	-.19**	—	
9. School level	-.22***	.16*	.03	-.04	.05	.13	.14*	-.43***	—
Secure profile									
1. Personal distress	—								
2. Empathic concern	-.27**	—							
3. Perspective taking	-.27**	.30***	—						
4. Irritability	.12	.07	-.27**	—					
5. Rumination/Dissipation	-.03	.12	-.03	.32***	—				
6. Gender	.18*	-.06	-.02	-.63***	-.21*	—			
7. Age	-.08	.03	.09	-.12	.18*	-.11	—		
8. Marital status	-.17	-.06	-.10	-.01	-.06	.09	.15	—	
9. School level	.13	-.07	-.13	-.30***	.08	.25**	.22*	-.16	—
Fearful profile									
1. Personal distress	—								
2. Empathic concern	.06	—							
3. Perspective taking	-.12	.17	—						
4. Irritability	-.01	-.05	.16	—					
5. Rumination/Dissipation	.10	.05	.06	.27**	—				
6. Gender	.20*	-.12	-.18	-.25**	-.24*	—			
7. Age	.00	.15	-.01	.12	.13	-.50***	—		
8. Marital status	-.15	-.20*	.02	.28**	.44***	-.13	-.22*	—	
9. School level	.04	.07	-.11	.00	-.20*	-.14	.05	-.45***	—
Dismissing profile									
1. Personal distress	—								
2. Empathic concern	-.23*	—							
3. Perspective taking	-.29**	.29**	—						
4. Irritability	.05	.01	.12	—					
5. Rumination/Dissipation	.10	-.05	.14	.26*	—				
6. Gender	.19	.02	-.18	-.05	-.04	—			
7. Age	-.12	.02	.32**	.32**	.13	-.47***	—		
8. Marital status	.13	.03	-.10	.02	.43***	-.04	.21*	—	
9. School level	-.18	-.15	-.11	-.58***	-.50***	-.12	-.33**	-.39***	—

*p < .05. **p < .01. ***p < .001.

## Discussion

The current study was aimed at contributing to the knowledge concerning processes leading to aggression. Our first aim was to analyse the link between empathy and aggression, starting from the contrasting results found in the literature and trying to clarify them by introducing the possible explaining and moderating role of attachment style. The data substantially confirmed our hypothesis. When considering the association between the two constructs, higher levels of empathy were linked to lower levels of aggression. This result appears in line with the traditional idea that sharing others’ internal state and vicariously experiencing their distress would encourage supportive behaviours, and discourage aggressive behaviours (Vachon et al., 2014). However, the introduction of the attachment style profile as a predictive variable of both empathy and aggression made their association non-significant, supporting the assumption that the negative relation between empathy and aggression might be accounted for by the underlying role of the attachment style. Results highlighted that, generally, securely attached individuals show higher empathy and lower aggressiveness than insecure individuals regardless of their type of insecure attachment style (preoccupied, fearful, or dismissing). Within insecurely attached individuals, no differences in the level of empathy were found, whereas fearful attached individuals showed higher aggressiveness than the preoccupied and dismissing individuals. This finding may be explained in light of the negative model of self and others that characterize the fearful individuals, which has the consequence of producing the need for keeping the others at distance, even using aggressiveness, to avoid intimacy and rejection episodes (Péloquin et al., 2011)

Furthermore, to better understand the potential mechanism through which attachment may influence the relation between empathy and aggression, we examined how different attachment style profiles may moderate their association, considering two general groups of secure and insecure people, but also distinguishing among specific groups of insecurely attached individuals, namely preoccupied, fearful and dismissing. Results showed that the secure attachment style group was characterized by a negative association between empathy and aggression, but no significant association was evidenced in the general or specific insecure groups. This difference could be a consequence of how generally insecure people feel both concerning self and others: negative models of self and/or others could involve feelings of discomfort toward others, as well as mixed feelings of need for approval and fear of being abandoned; this might be related to greater levels of aggressive behaviour to avoid potential close relationships or rejection events and could make it difficult to understand the thoughts and the real intentions of others. According to Mikulincer and Shaver (2007), individuals with a secure attachment may easily draw on the entirety of their psychological resources to maintain positive relationships; on the contrary, people with insecure attachment may be completely absorbed by their needs, consuming those attentional and caring resources that can be addressed to others. Consequently, empathy appears to be a well-rooted resource for securely attached individuals in deterring aggressive behaviour, but not for insecure people.

Additionally, attachment theory suggests that secure individuals, who can trust, seek advice from, and communicate openly with others may show better reactions than insecure individuals when experiencing others’ emotional states. Security promotes emotion regulation, a key capacity underlying empathic concern, and it is more likely that, when exposed to others’ feelings, secure individuals can vicariously feel them, without distress and anxiety, so reducing their aggressive tendency (Calkins & Leerkes, 2011; Eisenberg, 2000). Contrariwise, insecure individuals are more likely to lack coping skills and abilities to regulate their emotions when exposed to other emotional states: for example, they might feel distressed and their empathic ability would not be able to limit their aggression tendencies, probably related to high personal physiological arousal and hyperactivation of emotionality (Stern & Cassidy, 2018).

As mentioned, the present research provides a contribution that opens new perspectives in the existing literature about the relationship between empathy and aggression, but it is not without limits. First, the research design limits the generalizability of the results, given that it is a cross-sectional study. Longitudinal studies would have allowed obtaining more conclusive results both in terms of cause and effect and in terms of long-term consequences of attachment style on the relationships between empathy and aggression.

Furthermore, the study was based only on self-report measures, and this may have produced an overestimation/underestimation of the studied relationships. In this line, Vachon et al. (2014) suggested that the study of the relation between empathy and aggression would be more valid if direct detection tools, such as observation, were used. Additionally, even if we forced cluster analysis on ASQ scores to obtain theoretically guided insecure attachment styles, our findings seem to suggest that ASQ is more suitable for pondering only a general group of insecurely attached adults (e.g., Fuchshuber et al., 2018). Maybe, the use of more discriminant measures, such as the Adult Attachment Interview (George, Kaplan, & Main, 1985), would have given us the chance to detect specific differences in aggression management within insecurely attached individuals, which have not emerged in our study. Considering these differences would be one of the most interesting advancements in future research.

Despite these limitations, our study has practical implications. It meets the urgent need for both new comprehensive knowledge of the psychological processes involved in reducing/exacerbating aggression and the development of empirically informed strategies to guarantee more successful prevention programmes (Moeller, 2001). The improvement of empathic skills is often an essential part of educational programmes for reducing aggressive and antisocial behaviour. However, these interventions often neglect the relevance of other aspects and personal characteristics. The awareness that attachment style, whose foundations are structured in early childhood, can intervene in explaining and moderating the effect of empathy on aggression would facilitate the development of more wide-ranging programmes aimed at promoting not only the construction of positive attachment relationships starting from childhood but also a work of de-structuring the dysfunctional internal working models toward re-structuring new and more functional ones. In this line, specific support interventions based on strengthening the personal abilities to manage emotions and relationships, rather than actions aimed at repairing aggressive behaviours, would be crucial to improve the quality of life of individuals with fewer psychological resources, such as those insecurely attached.
